# A case report: Castleman disease treated by the trinity of internal and external treatment of “Fuzheng, phlegm-resolving, and detoxification method”

**DOI:** 10.1097/MD.0000000000037110

**Published:** 2023-02-02

**Authors:** Xinbei Yuan, Hua Fu, Min Xu, Wei Shen, Wenyi Zhou, Xi Li, Xinjin Gan

**Affiliations:** a Longhua Hospital, Shanghai University of Traditional Chinese Medicine, Shanghai, China.

**Keywords:** case report, Castleman disease, external treatment of traditional Chinese medicine, traditional Chinese medicine

## Abstract

**Rationale:**

Castleman disease (CD) was first included in the CSCO lymphoma diagnosis and treatment guidelines in 2021. Its diagnosis relies on lymph node pathological examination. Observation, surgical resection of the lesion, radiotherapy, chemotherapy, and medical therapy (e.g., rituximab, siltuximab, steroids) can be used. Due to the traumatic, incurable, and recurrent nature of surgical treatment, drug therapy has many side effects and is expensive. Exploring effective traditional Chinese medicine (TCM) comprehensive treatment methods for this disease is important and necessary.

**Patient concerns:**

The main symptom was recurrent lymphadenopathy, which had been surgically removed 5 times in the past. This time, lymph node enlargement occurred again, and the local hospital recommended surgical resection again. The patient could not tolerate another surgical treatment. Other targeted treatments are not available due to financial constraints.

**Diagnoses:**

The case was diagnosed as CD by pathological examination, which is an important basis for the diagnosis of this disease.

**Interventions:**

The patient was treated with surgery in the early stage several times, later came to our hospital for the trinity of TCM integrated treatment program, which combines oral TCM with external application of TCM and intravenous drip of TCM as a syndrome of positive deficiency and phlegm-toxin internalization, and the therapeutic principle.

**Outcomes:**

After nearly 3 years of comprehensive treatment with TCM, the enlarged lymph nodes could not be touched, and there was no fatigue, fever, or weight loss. During this period, the patient did not undergo surgery, chemotherapy and other western medicine treatment, and lived a normal life. It not only met the patient’s expectation but also confirmed that the TCM treatment was indeed effective.

**Lessons:**

This case report confirms that TCM is safe and effective in the treatment of CD, which is worthy of promotion. In clinical practice, the individualized treatment for the patient, the duration of treatment, and the different disease states also affect the treatment outcome.

## 1. Introduction

Castleman disease (CD), also known as giant lymph node hyperplasia or angiofollicular lymph node hyperplasia, is a highly heterogeneous clinicopathological entity belonging to the family of lymphoproliferative disorders, and it was listed in the first batch of Chinese rare disease catalog in 2018.^[1]^ Its main clinical manifestation is enlargement of one or more lymph nodes, and a few patients may be accompanied by lymph node compression symptoms and systemic symptoms (such as fever, night sweats, emaciation, anemia, etc). The diagnosis of this disease mainly relies on lymph node pathologic examination. Depending on the type of pathology, degree of lymph node involvement and clinical presentation, different treatments may be used, such as observation, complete surgical resection of the lesion, radiotherapy, chemotherapy and monoclonal antibody (e.g., cetuximab and rituximab)-based therapies.^[2]^

Among the aforementioned treatments, radiotherapy, chemotherapy, surgery, and monoclonal antibody therapy play an important role in the treatment of CD, but radiotherapy has many complications and even leads to partial loss of function, surgical resection has a high recurrence rate, chemotherapeutic drugs have high toxicity and side effects, and monoclonal antibody therapy is expensive. Therefore, exploring effective traditional Chinese medicine (TCM) comprehensive treatment methods for this disease is important and necessary.

There is currently no standard treatment or experience summary for Castleman disease in TCM, and very few case reports are available.^[3,4]^ Summarizing the experience^[5–7]^ of TCM in the treatment of malignant lymphoma and relevant diagnosis and treatment guidelines,^[8]^ we classify CD into the category of “malignant nucleus,” which is often identified as a syndrome of positive deficiency and phlegm-toxin internalization, and the therapeutic principle is to support the correctness of phlegm, resolve phlegm, and detoxify toxin. Dr Gan Xinjin of the Department of Hematology of Longhua Hospital formulated a trinity TCM comprehensive treatment method of “supporting righteousness, resolving phlegm, and detoxification’’ by identifying and using the comprehensive treatment of Chinese medicine oral, intravenous injection and external application of Chinese medicine. This method of treatment for Castleman disease has achieved remarkable clinical efficacy. Therefore, a case report is shared here for the benefit of readers.

## 2. Case report

Mr. Wan, male, 56 years old, was in good health and had no family hereditary diseases, presented with enlarged left cervical lymph nodes in March 2013 without obvious triggers. Initially, the lymph node was soft in texture with no pressure pain and elevated skin temperature accompanied by night sweats, but it was not taken seriously. Subsequently, the mass increased in size and was mistaken for lymphoma or lymphadenitis at first due to the rarity of the disease. Then, he underwent left cervical lymph node aspiration in October 2013,confirmed the diagnosis of Castleman disease, plasma cell type, based on lymphoproliferative disorders and immunohistochemical markers. He subsequently underwent surgical excision treatment to remove a 4-cm mass. After discharge from the hospital, his night sweats improved, but the left neck mass continued to grow, and he underwent 5 surgical resections from 2015 to early 2019 with similar pathologic findings.

In April 2019, Mr. Wan left cervical lymph node became enlarged again, and on September 5, 2019, he was initially seen in Dr Gan Xinjin clinic. The superficial lymph node B-scan ultrasonography showed that the left posterior cervical lymph node was enlarged (30*8 mm hypoechoic mass with a small number of lymphatic vessels was seen in the left posterior cervical region). His blood routine, liver and kidney functions were normal. The patient had a hard fixed mass about 3*2 cm in size in the left neck, which was painless to touch and had no change in skin color. Carved symptoms: the patient has no fever, no emaciation, occasional night sweats, poor appetite, normal diet, normal bowel movements, normal sleep, red tongue with thin fur, and stringy pulse. Distinguish the disease “Malignant nucleus disease,” and differentiate the syndrome as “positive deficiency and toxic junction syndrome.” The treatment methods are fuzheng phlegm, detoxification and sanjie, Fuzheng Huatan Jiedu prescription is added or subtracted as follows:

Ripe rehmannia 15 g, Astragalus 15 g, Polygonati Rhizoma 12 g, Ganoderma 9 g, Fritillaria thunbergii Miq. 9 g, Sinapis Semen 9 g, Cassia cinnamon 3 g, Oldenlandia diffusa 30 g, Amorphophallus rivieri Durieu 15 g, licorice 3 g. One dose per day, totaling 300 mL, twice in the morning and evening, half an hour after meals. Compound matrine injection and other intravenous drip, once a day for 7 days, and the self-made “Tumor-Flattening Ointment” external application, 4 hours a day, in order to dissolve phlegm and disperse the knot.

Thereafter, the patient was seen regularly and the herbal prescription was adjusted according to his condition with daily application of herbal medicine combined with “Tumor-Flattening Ointment” and combined with intravenous infusion in our department once every 3 months.

Prior to this case report, an enhanced CT of the patient’s neck at his last visit in July 2022 showed multiple cervical lymph nodes (the larger one was approximately 6.8 mm in diameter and showed heterogeneous enhancement changes on enhancement). His blood counts, liver and renal functions were normal. The patient still had no fatigue, no fever, no emaciation, normal diet, normal bowel movement, normal sleep, red tongue with thin fur, and stringy pulse. The treatment proposed was still mainly to fuzheng phlegm, detoxification and dispersing knot. Due to the shrinkage of the mass, the prescription was adjusted, and the decoction method was the same as before, with ephedrine injection, etc. Since the superficial mass was not palpable, the external treatment method was stopped.

Between September 2019 and July 2022, the patient underwent regular superficial lymph node follow-up examinations and enhanced CT scans to assess changes in lesion size. The superficial B-ultrasound results are shown in Table 1, and the neck-enhancing CT results are shown in Table 2.

**Table 1:** Superficial B-ultrasound results of lymph node enlargement.

Examination date	Lymph node location	Size (mm) and quantity	Morphology
2019/9/9	Left posterior cervical lymph node	Several low echogenicity, 30*8, 19*10	A small amount of lymphatic portal
2020/9/18	Bilateral cervical lymph nodes	Several low echogenicity, one of which is 11*6	Lymphatic portal visible
2021/11/2	Slightly enlarged left submandibular lymph node	Low echogenicity, 16*5.4 low echogenicity	Lymphatic portal visible
2022/7/26	No obvious enlarged lymph nodes in the bilateral neck, clavicle, bilateral groin, and bilateral axilla		

**Table 2:** Enhanced CT results of lymph node enlargement in the neck.

Examination date	Conclusion of neck enhanced CT	Size
2020/5/6	Multiple lymph nodes were enlarged in the left chest, clavicle, submandibular gland area, and below	Maximum diameter of approximately 16mm
2021/11/1	Multiple lymph nodes were enlarged in the left chest, clavicle, submandibular gland area, and below, with some slightly enlarged compared to the image taken on 2021-06-28, and slightly reduced in size.	The maximum diameter is approximately 10mm
2022/7/27	Multiple lymph nodes were enlarged in the left chest, clavicle, submandibular gland area, and upper neck on both sides, which were roughly similar to those on 2022-02-21	The short diameter of the larger one is approximately 6.8 mm

**Figure 1.**
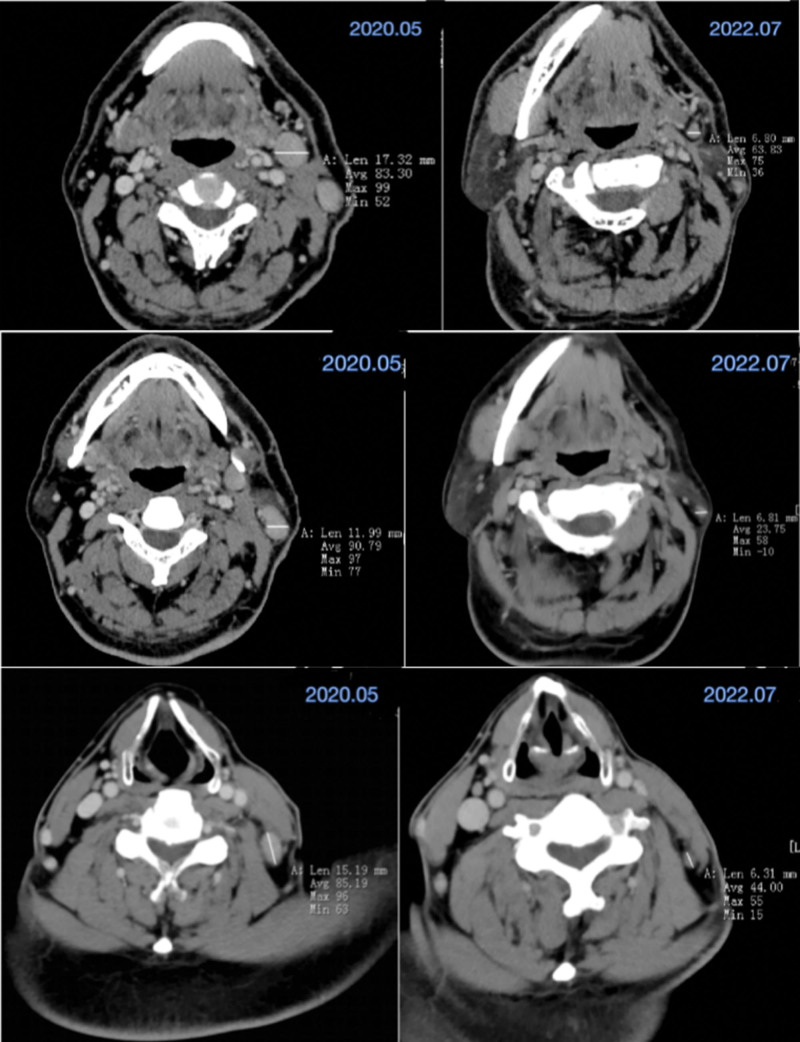
CT images show changes in lymph node size at different levels before and after treatment.

From the above table (Tables 1 and 2) and CT images (Fig. 1), it can be seen that the patient’s enlarged lymph nodes gradually decreased in size during the period of comprehensive treatment with herbal medicine.

Thus far, the patient has received nearly 3 years of comprehensive Chinese medicine treatment, including regular oral intake of Chinese medicine decoction, regular intravenous infusion of Chinese medicine, and external application of “Tumor- Flattening Ointment.” During this period, no western medical treatment such as surgery or chemotherapy has been performed, and the patient’s quality of life has been good. According to the CDCN 2017 version of efficacy evaluation criteria, the patient’s hemoglobin, C-reactive protein, albumin, and creatinine clearance were normal, and there were no symptoms such as fatigue, anorexia, fever, and emaciation. Lymph nodes were assessed according to the Cheson criteria, and no lymph nodes were palpable, reaching CR, indicating that the patient’s overall efficacy had reached complete remission. The patient’s pretreatment expectations were met and he was willing to recommend other patients to try TCM treatment.

## 3. Discussion

### 3.1. Dialectical treatment

The diagnosis and treatment of Castleman disease have not been described in detail in ancient and modern Chinese literature. The main clinical manifestations are enlargement of one or more lymph nodes, and a few are accompanied by symptoms of lymph node compression or systemic symptoms such as fever, night sweats, emaciation and anemia. Based on the clinical manifestations of the disease, it can be categorized into “malignant nuclear disease” and other disease patterns. As early as in the book of “Surgery Complete Treatment of Life,” it was said that “the big one is called malignant nucleus, and the small one is called phlegm nucleus. However, the cold condensation poison root is the deepest, but not easy to ulcerate,” explaining the pathogenesis of “Malignant nucleus disease.” There are only 2 domestic reports on the effective treatment of Castleman disease by TCM. It is believed that the deficiency of Qi and Yin and the condensation of phlegm and heat are the main causes of the disease, and the treatment is to clear heat, transform phlegm, disperse nodules, and tonify Qi and nourish Yin.^[1]^ Alternatively, it is believed that the disease is caused by the deficiency of lung and kidney Qi and Yin. When Yin is deficient, the fire is strong, and the heat burns the fluid into phlegm. The phlegm and heat condense each other, so the treatment is to tonify the lungs and kidneys, activate blood and open collaterals, and clear heat and disperse nodules.^[2]^ Based on the clinical manifestations and pathogenesis of Castleman disease, our department identified it as “Malignant nucleus disease,” which belongs to the disease of deficiency of the essence and mixed deficiency and reality. Therefore, the principle of treatment is to tonify the positive in order to help the evil, and to resolve the phlegm and detoxify the poison in order to get rid of the evil.

### 3.2. Medicine for disease

If the condition is recurring and the patient has no underlying disease, the focus should be on attacking the pathogen, with the addition of medicines such as Amorphophallus rivieri Durieu, Oldenlandia diffusa, Sophorae Flavescentis Radix, and Duchesnea indica, in order to strengthen the effect of clearing heat and detoxification, antitumor, and to slow down the progression of the disease.

Amorphophallus rivieri Durieu^[9]^ has the functions of resolving phlegm, dispersing stasis, and reducing swelling. Its antitumor mechanism may be related to the inhibition of tumor cell proliferation, enhancement of cellular immunity, cytotoxicity.^[10]^ It has found that TuAKe can inhibit the proliferation of human chronic myeloid leukemia K562 cells and induce the multidirectional differentiation of K562 cells, thus possessing antitumor effects.^[11]^

Oldenlandia diffusa^[12]^ can act directly on tumor cells, and high doses of DPE have significant antitumor effects (similar to cyclophosphamide). The Sophorae Flavescentis Radix^[13]^ has a high content of flavonoids with cytotoxic and antitumor effects.

### 3.3. Intravenous medication

Infusion therapy played an important role in medicine and gradually took shape in the 17th and 18th centuries. Most intravenous preparations were chemical compounds. Chinese herbal medicine has a history of thousands of years in China, and the earliest TCM injection was established in 1941. With the development of scientific research, the improvement of standards and standardization, more TCM injections have been used in clinical practice in recent years. On the basis of symptomatic typing and disease-specific medication, combined with the development of modern medicine, we often use drugs such as matrine injection for complementary treatment. Studies have shown that Kushen injection has antitumor effects, especially when combined with chemotherapy drugs.^[14,15]^ The combination of matrine injection and R-CHOP chemotherapy for the treatment of non-Hodgkin lymphoma can induce down-regulation of Bcl-2 gene expression with fewer adverse effects.^[16]^ In addition, the study also found that TCM injections can improve the clinical efficacy and control rate of non-Hodgkin lymphoma patients to varying degrees.^[17,18]^ The application of antitumor TCM injection is based on the disease characteristics of Castleman disease and lymphoma.

### 3.4. Traditional Chinese medicine external treatment

**Figure 2.**
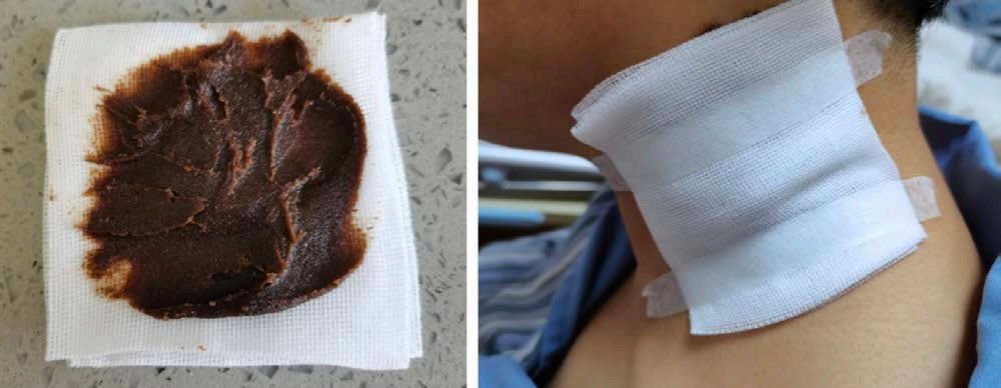
The picture shows the “Tumor-Flattening Ointment” and the patient is being treated.

The external treatment method of Chinese medicine has a long history. As early as in the book of “Suwen,” it was mentioned that “internal diseases are treated internally, and external diseases are treated externally.” External application of medicine for the treatment of enlarged lymph nodes has been widely used from ancient times to the present. According to literature, the use of Xiaoluo San^[19]^ for the treatment of malignant lymph node enlargement resulted in an overall effective rate of 90.5% in relieving lymph node swelling and pain. The total effective rate of LuoLi Xiao^[20]^ in treating cervical lymph node enlargement was 85.00%. The Department of Hematology of Longhua Hospital has been making and using Tumor-Flattening Ointment to treat superficial tumors caused by lymphoma and other diseases since 2011. The formula has been optimized year by year based on clinical experience and efficacy. The current composition includes cinnamon, Prunella vulgaris, Acorus tatarinowii Vaccariae Semen, Sinapis Semen, Bufo melanostictus, and Curcuma zedoaria. Among them, cinnamon is spicy and hot, which can dissolve cold phlegm. It is often used t in combination with Chinese herbs with softening and dispersing effects, such as Vaccariae Semen, Sinapis Semen, Prunella vulgaris, Acorus tatarinowii, and so on. The antitumor effect is exerted by adding heat-clearing, and detoxifying TCM. Meanwhile, it is believed that the formation of tumor masses is often the result of phlegm and blood stasis. Therefore, blood-activating and stasis-resolving herbs such as Curcuma zedoaria are added to achieve the effect of warming and dissolving phlegm and blood stasis, softening hard masses, and dispersing stagnation. In this case, the patient used “Tumor-Flattening Ointment” externally, and the topical medication penetrated into the skin and reached the affected area directly, like Fig. 2.

## 4. Conclusion

Castleman disease is classified as a rare disease, and there are few reports about its TCM treatment.

Its clinical manifestations have certain similarities with malignant lymphoma, for which a variety of TCM therapies, such as oral decoction, proprietary Chinese medicines, topical application, and acupuncture, have been included in the National Health Commission’s “Guidelines for the Diagnosis and Treatment of Lymphoma (2018 Edition).” Based on the TCM comprehensive therapy of “strengthening the body, resolving phlegm, and detoxification,” which has been clinically applied for many years, the combination of local and systemic diagnosis and treatment, and the combination of internal and external treatment, and giving full play to the advantages of Chinese medicine, can improve the clinical symptoms of the patients and improve the quality of life, without obvious adverse and unanticipated events.

The patient in this case was diagnosed as Castleman disease by pathology, and the disease recurred after multiple surgical resections. Through the comprehensive treatment of TCM identification and typing, internal and external combination, the imaging evaluation showed remarkable efficacy. This fully demonstrates the advantages of TCM in treating this disease and deepens the exploration of the treasure trove of TCM. At the same time, it is imperative to explore the TCM integrated treatment for other hematologic and rare diseases.

## Author contributions

**Formal analysis:** Xi Li.

**Methodology:** Min Xu, Wei Shen.

**Supervision:** Xinjin Gan.

**Resources:** Wenyi Zhou.

**Writing – original draft:** Xinbei Yuan.

**Writing – review & editing:** Hua Fu, Xinjin Gan.
